# Improved estimation of glomerular filtration rate (GFR) by comparison of eGFR_cystatin C_ and eGFR_creatinine_

**DOI:** 10.3109/00365513.2011.634023

**Published:** 2011-11-28

**Authors:** Anders Grubb, Ulf Nyman, Jonas Björk

**Affiliations:** 1 Department of Clinical Chemistry, Lund University Hospital, Lund, Sweden; 2 Department of Radiology, University of Lund, Lasarettet Trelleborg, Trelleborg, Sweden; 3 Competence Centre for Clinical Research, Lund University Hospital, Lund, Sweden

**Keywords:** Kidney function, immunoassays, kidney diseases, cystatin C, creatinine

## Abstract

**Objective:**

GFR-prediction equations based upon cystatin C and creatinine have better diagnostic performance in estimating GFR than equations based upon only one of the two markers. The present work concerns in what way a comparison between separate estimations of GFR based upon cystatin C (eGFR_cystatin C_) or creatinine (eGFR_creatinine_) can be used to evaluate the diagnostic performance of a combined cystatin C- and creatinine-based estimation of GFR.

**Methods:**

The difference between eGFR_cystatin C_ and eGFR_creatinine_ was compared with measured GFR (iohexol clearance) and a combined cystatin C- and creatinine-based estimation of GFR in a Swedish-Caucasian cohort of 857 adult patients.

**Results:**

A difference between eGFR _cystatin C_ and eGFR_creatinine_ of ≥ 40% indicated a markedly reduced diagnostic performance of the combined cystatin C- and creatinine-based estimation of GFR.

**Conclusion:**

Comparison of the agreement between eGFR_cystatin C_ and eGFR_creatinine_ can be used to evaluate the diagnostic performance of combined cystatin C- and creatinine-based estimations of GFR. If ‘threshold values’ for discordance are exceeded, it must be considered whether the clinical context requires the use of an invasive gold standard method to measure GFR. In some clinical contexts either creatinine or cystatin C are known to be invalidated as markers of GFR and in these situations the use of only the cystatin C- or the creatinine-based GFR estimate should be considered when the ‘threshold values’ are exceeded.

## Introduction

GFR-prediction equations based upon cystatin C (eGFR_cystatin C_) or creatinine (eGFR_creatinine_) may produce estimated GFR-values, of which 80–85% are within ± 30% of GFR measured by invasive gold standard methods. However, the highest percentages of estimated GFR-values within ± 30% of measured GFR are obtained using GFR-prediction equations based upon both cystatin C and creatinine (eGFR cystatin C + creatinine [1-9]. The performance of eGFR_creatinine_ is reduced *inter alia* if a patient has an abnormally low or high muscle mass, recently ingested boiled meat or is treated with a drug that influences the tubular secretion of creatinine. The performance of eGFR_cystatin C_ is reduced *inter alia* if a patient is treated with large doses of glucocorticoids. In such clinical situations the diagnostic performance of a GFR-prediction equation based upon both cystatin C and creatinine may be inferior to those equations based upon only one of the GFR-markers [1]. See also www.egfr.se. However, such situations may not always be recognized by those ordering a GFR-estimate or by the laboratory performing the tests. It has therefore been suggested that a comparison between separate estimations of GFR based upon cystatin C or creatinine can be used to evaluate the diagnostic performance of a combined cystatin C- and creatinine-based estimation of GFR [1]. The present work concerns the relation between the agreement between eGFR _cystatin C_ and eGFR _creatinine_ and the diagnostic performance of eGFR _cystatin C + creatinine_.

## Material and methods

The patient population studied was identical to the one previously used to analyse various equations to combine creatinine and cystatin C to predict GFR [8]. It consisted of adult patients (Swedish-Caucasians ≥ 18 years) consecutively referred to the Departments of Clinical Chemistry, University Hospitals of Lund and Malmö for determination of GFR by iohexol clearance. Simultaneous measurements of plasma creatinine, plasma cystatin C, weight and height were performed and age and gender recorded.

The Lund population consisted of 451 patients (225 females) and the Malmö population of 425 patients of whom 19 patients were excluded because of missing plasma creatinine values (*n* = 6), missing plasma cystatin C values (*n* = 8) or technical assay errors (*n* = 5) leaving 406 subjects in the Malmö cohort. All procedures involving subjects and data were in accordance with the Helsinki Declaration of 1975 concerning ethical principles for medical research involving human subjects.

The characteristics of the two cohorts and the combined set (*n* = 857) are shown in Table I and included 12 patients with neurological diseases and secondary muscular atrophy. Common indications for referral were diagnosis and follow-up of chronic kidney disease, evaluation of renal function prior to dosage of drugs cleared by the kidneys, evaluation of potential renal donors, follow-up of unilaterally nephrectomized patients, pre-operative evaluation of patients with hyperparathyroidism and control of renal transplants (*n* = 44).

**Table I:** Demographic and anthropometric patient characteristics, plasma creatinine, plasma cystatin C, and iohexol clearance given as median values (2.5 and 97.5 percentiles) in the Lund and Malmö cohorts as well as in the combined set.

Parameters	Lund (*n* = 451)	Malmö (*n* = 406)	Combined (*n* = 857)
Age (years)	58 (24–83)	61 (26–85)	59 (26–85)
Females	225 (50%)	152 (37%)	377 (44%)
Total body weight (kg)	73 (46–115)	78 (49–111)	75 (48–112)
Height (cm)	170 (151–189)	173 (152–190)	171 (152–189)
Body surface area (m^2^)	1.83 (1.45–2.31)	1.92 (1.49–2.33)	1.88 (1.46–2.31)
Body mass index (kg/m^2^)	25 (18–39)	26 (18–38)	25 (18–38)
Plasma creatinine (μmol/L)	92 (39–400)	136 (54–623)	106 (44–545)
Cystatin C (mg/L)	1.18 (0.79–3.07)	1.53 (0.85–4.06)	1.30 (0.81–3.82)
Iohexol clearance (mL/min per 1.73 m^2^)	63 (11–124)	42 (8–115)	55 (9–121)

### Determination of iohexol clearance

Five mL of iohexol (Omnipaque 300 mg iodine/mL, GE Healthcare, Oslo, Norway) were administered intravenously in an antecubital vein. Iohexol clearance (referred to as ‘measured GFR') was calculated from plasma clearance of a single plasma sample of iohexol [10] drawn at varying times, normally 4 hours after injection, according to expected GFR as determined by plasma creatinine concentration and anthropometric data. The exact time of administration and blood sampling were documented by a specialist nurse. Plasma iohexol concentrations were determined by high-pressure liquid chromatography with a total analytical variation of 2–4% (coefficient of variation, CV%) at the range of iohexol concentrations normally encountered during the study [11]. The Dubois formula was used to adjust the measured GFR values to 1.73 m^2^ body surface area [12].

### Determination of plasma creatinine

Plasma concentrations of creatinine were determined at Lund University Hospital by an enzymatic colorimetric assay on a Hitachi Modular P analyzer (Roche Diagnostics, Mannheim, Germany) and with a calibrator traceable to primary reference material with values assigned by isotope dilution mass spectrometry (IDMS) [13]. At Malmö University Hospital a modified Jaffe colorimetric method was used on a Beckman LX20 analyzer (Beckman Coulter, Inc., Fullerton, CA, USA) employing zero-point calibration and a calibrator traceable to primary reference material with values assigned by IDMS [14,15]. Total analytical variation (CV%) of the enzymatic method in Lund was 1.4–3.0% at concentrations of creatinine between 60 and 578 μmol/L and 2.2–2.8% at concentrations between 53 and 631 μmol/L for the Jaffe method in Malmö.

### Determination of plasma cystatin C

Plasma cystatin C levels were determined by an automated particle-enhanced immunoturbidimetric method [16] using a Hitachi Modular P analysis system, reagents (code Nos LX002, S2361, X0973, X0974) obtained from DakoCytomation (Glostrup, Denmark) and following the procedure recommended by the reagent producer. The procedure had a total coefficient of variation of 2.1% at a cystatin C level of 1.0 mg/L and of 1.7% at a level of 4.0 mg/L. All samples were analysed within one day after collection or frozen at −20°C until analysed.

### Prediction equations

The Lund-Malmö creatinine-based equation (eGFR_creatinine_) with age and gender [17] and the Grubb cystatin C-based equation (eGFR_cystatin C_) based on adults and including gender [2] were selected for the present analysis. Plasma creatinine (pCr) is expressed in μmol/L, plasma cystatin C (pCy) in mg/L, age in years and ln denotes the natural logarithm. Both equations express relative GFR in mL/min per 1.73 m^2^ body surface area.

### Lund-Malmö creatinine equation (eGFR_creatinine_)

GFR = e^X − 0.0124 × age + 0.339 × ln(age) − 0.226 (if female)^ X = 4.62 − 0.0112 × pCr (if pCr < 150 μmol/L) X = 8.17 + 0.0005 × pCr − 1.07 × ln(pCr) (if pCr ≥ 150 μmol/L)

### Grubb cystatin C equation (eGFR_cystatin C_)

GFR = 86.49 × pCy^−1.686^ × 0.948 (if female), equivalent with GFR = e^4.46 − 1.686 × ln (pCy) − 0.053 (if female)^

GFR estimates from the combined use of the two analytes (eGFR_cystatin C__+__creatinine_) were based on the arithmetic mean of eGFR_cystatin C_ and eGFR_creatinine_, which has proved as accurate as more complex equations [8].

### Statistical evaluation

All statistical analyses were conducted using SPSS release 18.0.1. (SPSS Inc, Chicago, USA). In the statistical testing we regarded *p*-values in the order of 0.05 as moderate evidence against the null hypothesis, whereas *p*-values in the order of 0.001 or below were regarded as strong evidence against the null hypothesis [18]. The present study focused on the *accuracy* of the arithmetic mean of eGFR_cystatin C_ and eGFR _creatinine,_ denoted eGFR_cystatin C + creatinine_, in relation to the *agreement* between eGFR_cystatin C_ and eGFR_creatinine_. The *accuracy* of eGFR_cystatin C + creatinine_ was reflected by the absolute percentage error:

|eGFR_cystatin C + creatinine_ − measured GFR| /measured GFR, and summarized as the percentage of estimates within 30% (P_30_) and 10% (P_10_) of measured GFR [19]. The *agreement* was reflected by the difference%, i.e. the absolute difference |eGFR_cystatin C_ − eGFR_creatinine_| expressed in percent relative to the arithmetic mean eGFR _cystatin C + creatinine_.

The following analyses were made:

1. Pearson's and Spearman's correlation coefficients (denoted r and r_s_) were used to evaluate the overall association between accuracy (absolute percentage error) and agreement (difference%).
2. The accuracy categorized as P_30_ and P_10_ was evaluated in relation to agreement (difference%) rounded to nearest integer and then categorized as < 10%, 10–19%, 20–29%, 30–39% and ≥40% difference. Fisher's exact test was used to evaluate differences in P_30_ and P_10_ across categories of agreement.
3. Measured GFR is related to both accuracy and agreement and may thus confound the association between agreement and accuracy. To account for such confounding we modelled accuracy, i.e. P_30_ and P_10_, respectively, using logistic regression with measured GFR and difference% as continuous covariates.
4. We calculated an ‘improvement index', defined as the proportion of all GFR estimates for which eGFR_cystatin C + creatinine_, but not both eGFR_cystatin C_ and eGFR_creatinin_, were inaccurate according to P_30_ and P_10_, respectively. This index represents the upper limit of the improvement in accuracy that could be obtained if the most accurate eGFR (i.e. eGFR _cystatin C + creatinine_, eGFR _cystatin C_ or eGFR _creatinin_) was consistently applied for each patient. Note that the sum of, e.g. P_30_ and the corresponding improvement index for P_30_ can never exceed 100%.
5. The improvement index depends on the accuracy, i.e. the potential for improvement is higher when accuracy is low. To account for such confounding in the association between agreement and the improvement index, we modelled the improvement index using logistic regression with inaccuracy (absolute percentage error) and difference% as continuous covariates.

## Results

The overall association between difference% and absolute percentage error was not consistent (r = 0.13, *p*<0.001 but r_s_ = 0.05, *p* = 0.12), however, P_30_ was clearly decreased for differences between eGFR_cystatin C_ and eGFR_creatinin_ exceeding a ‘threshold value’ of 40% (Table II; *p* = 0.02 when comparing P_30_ for 30–39 and >40% difference). The dip in accuracy when expressed as P_10_ seemed to occur already at 30–39% difference, but the statistical evidence for this dip was weak (*p* = 0.09 when comparing P_10_ for 20–29 and 30–39% difference). Measured GFR was noticeably lower for differences exceeding 40%, but the suggested inverse association between difference% and P_30_ remained clear when measured GFR was adjusted for using logistic regression (*p* = 0.001), whereas the association between difference% and P_10_ remained weaker (*p* = 0.05).

**Table II:** Accuracy of eGFR _cystatin C + creatinine_, the arithmetic mean of eGFR_cystatin C_ and eGFR_cretinine_, calculated as the percentage of estimates within 30% (P_30_) and 10% (P_10_) of measured GFR in relation to the difference% between eGFR_cystatin C_ and eGFR_creatinine_, defined as |eGFR_cystatin C_ − eGFR_creatinine_| / eGFR_cystatin C + creatinine_. The improvement index, defined as the proportion of all GFR estimates where eGFR_cystatin C + creatinine_, but not both eGFR_cystatin C_ and _creatinine_, were inaccurate within 30% and 10% of measured GFR, is also presented.

		Accuracy (%)		Improvement index (%)	
Difference %	Median measured GFR (mL/min per 1.73 m^2^)	30%	10%	30%	10%
<10 (*n* = 220)	59	90.0	43.6	1.8	8.2
10–19 (*n* = 200)	59	91.5	48.5	2.5	25.0
20–29 (*n* = 175)	61	94.3	45.1	5.7	39.4
30–39 (*n* = 120)	54	90.0	35.0	8.3	52.5
≥ 40 (*n* = 142)	39	79.6	40.8	18.3	30.3
Total (*n* = 857)	55	89.5	43.4	6.4	28.4

The improvement index generally suggested higher potential for improvement in accuracy when the difference between eGFR_cystatin C_ and eGFR_creatinin_ was considerable (Table II). This association between the agreement (difference%) and the potential for improvement in accuracy remained evident when differences in accuracy across levels of agreement were adjusted for using logistic regression (*p<*0.001 both for improvement in P_30_ and in P_10_).

## Discussion

The diagnostic performance of eGFR_creatinine_ is reduced *inter alia* if a patient has an abnormally low or high muscle mass, recently ingested boiled meat or is treated with a drug that influences the tubular secretion of creatinine. In these clinical contexts the diagnostic performance of eGFR_cystatin C_ is generally unaltered. However, the performance of eGFR _cystatin C_ is impaired if a patient is treated with large doses of glucocorticoids and in this situation the performance of eGFR_creatinine_ is still acceptable. Although GFR-prediction equations based upon both cystatin C and creatinine (eGFR_cystatin C + creatinine_) generally are superior to GFR-prediction equations based upon either cystatin C (eGFR_cystatin C_) or creatinine (eGFR_creatinine_) this may not be the case in these specific clinical contexts. Although these contexts may be easily recognized in some cases, they will not invariably be recognized. There may also be additional, not yet identified, clinical contexts invalidating either cystatin C or creatinine as useful markers for GFR. Comparing eGFR _cystatin C_ and eGFR_creatinine_ might be helpful to identify both known and unknown causes when neither cystatin C nor creatinine are suitable as a marker for GFR [1]. To be able to efficiently use such a comparison, it must be known when the discordance between eGFR_cystatin C_ and eGFR_creatinine_ is large enough to indicate such a condition. The present study based upon measured GFR and eGFR_cystatin C_ and eGFR_creatinine_ in a patient cohort of 857 Swedish-Caucasian adult patients indicates that if the discordance is 40% or more, the diagnostic performance of eGFR_cystatin C + creatinine_ is markedly reduced. Such discordance should initiate a more careful evaluation of the clinical context to disclose conditions invalidating either creatinine or cystatin C as a GFR marker. If such conditions are identified, GFR might be best estimated using a prediction equation based upon only the non-invalidated marker. If such conditions are not identified, it should be realized that the estimation of GFR is unreliable and that an invasive, gold standard, measurement of GFR might be required.

It should be realized, that the discordance value, the ‘threshold value', indicating requirement of a further evaluation of the clinical context to improve estimation of GFR, as presented in this work, is influenced by the actual patient cohort and by the equations used for estimating GFR, i.e. eGFR _cystatin C_, eGFR_creatinine_ and eGFR_cystatin C + creatinine_. For other patient cohorts might contain proportionally more, or fewer patients, with conditions invalidating either cystatin C or creatinine as a GFR marker. The more patients with such conditions in the cohort, the greater the potential for improvement of eGFR by comparison of eGFR_cystatin C_ and eGFR_creatinine_. The equations for eGFR_cystatin C_, eGFR_creatinine_ and eGFR_cystatin C + creatinine_ will also influence the ‘threshold value’ by being more or less sensitive for patient characteristics reducing the value of creatinine and cystatin C as markers for GFR.

It is possible to calculate an ‘improvement index', defined as the proportion of all GFR estimates for which eGFR_cystatin C + creatinine_, but not both eGFR_cystatin C_ and eGFR_creatinin_, are inaccurate according to P_30_ or P_10_. This index will represent the upper limit of improvement in accuracy that could be obtained, if the most accurate eGFR (i.e. eGFR_cystatin C + creatinine_, eGFR_cystatin C_ or eGFR_creatinin_) was consistently applied. In the present study the improvement index was 18.3 % for a discordance threshold of ≥40%, which means that if an accurate evaluation of the relevant clinical conditions could be performed for each patient the P_30_-value of 79.6% for eGFR_cystatin C + creatinine_, would theoretically increase to 79.6 + 18.3 = 97.9%. This ‘improvement index’ will, exactly like the ‘threshold value’ of discordance, also be influenced by the actual patient cohort and by the equations used for eGFR, i.e. eGFR_cystatin C_, eGFR_creatinine_ and eGFR_cystatin C + creatinine_ and for the same reasons.
